# BCR‐ABL exon 7 deletion and novel point mutation in patient with chronic myelogenous leukemia and TKI resistance

**DOI:** 10.1002/ccr3.1794

**Published:** 2018-09-14

**Authors:** Ilya Mikhailov

**Affiliations:** ^1^ Faculty of Fundamental Medicine Lomonosov Moscow State University Moscow Russian Federation

**Keywords:** BCR‐ABL, CML, deletion, point mutations

## Abstract

I report a novel BCR‐ABL point mutation c.844G>C p.E282Q and a case of combination of two BCR‐ABL point mutations (p.E282Q and p.L298R) and exon 7 deletion (del. c.1086‐1270) in TKI‐resistant patient. These point mutations were present only in a truncated transcript and were absent in “wild‐type” BCR‐ABL transcript.

## 1 INTRODUCTION

Chronic myelogenous leukemia is a myeloproliferative disorder which is characterized by malignant transformation of blood progenitor cells. BCR‐ABL fusion gene formation after reciprocal translocation t(9;22)(q34.1;q11.2) plays a crucial role in CML pathogenesis. BCR‐ABL‐protein is a constitutively active nonreceptor tyrosine kinase which induces the activation of some proliferative cascades.^1^ Modern CML treatment concept is based on BCR‐ABL tyrosine kinase inhibitors (TKI) but this strategy is associated with one major problem—after 1‐2 years of TKI therapy, some patients acquire secondary resistance which is mainly caused by appearance of kinase domain point mutations.^2^ Nevertheless, the pathogenic role of other resistance factors, for example deletions and insertions, is unclear and this question requires further studies. Here, I report a combination of novel point mutation and exon 7 deletion (del. c.1086‐1270) in a patient resistant to first and second TKI generations.

## 2 CASE REPORT

An 18‐year‐old woman was admitted to our center in 2000 with persistent general malaise and fever. Physical and ultrasound examination showed increased spleen size (+0.2 dm). Laboratory data showed significant leukocytosis (26.8 × 10^9^/μL) and basophilia (20%). According to these indicators, accelerated phase of CML has been diagnosed, after cytogenetic analysis Ph‐chromosome was detected in all bone marrow myeloid cells (BMC). After 6 months of chemotherapy with hydrea and idarubicin, patient has been receiving imatinib 400 mg/d the first month, 600 mg/d for 2 following months, and 800 mg/d for 8 following years. Significant cytogenetic response (20% Ph+ bone marrow cells) and optimal molecular response (BCR‐ABL/ABL ratio = 9.43%) were achieved by the end of the first year but cytogenetic (CyR) and molecular responses were lost after 18 months of treatment with imatinib (CyR = 43% Ph+ cells, BCR‐ABL/ABL = 51.77%). After 8 years of treatment, the hematologic response was lost (basophilia more than 20%), Ph‐chromosome was detected in 67% of cells, the BCR‐ABL/ABL ratio was 75.81%, and also cDNA direct sequencing revealed M351T mutation.

In 2009, bosutinib therapy had been started, and after the first month of treatment with 500 mg/d, cytogenetic response had been 55% Ph+ cells and molecular response had been 62.34%, but after 3 months, it was lost (CyR = 67%; BCR‐ABL/ABL = 88.44%) and the dosage was increased to 600 mg/d no effect.

In 2013, direct Sanger sequencing of cDNA revealed two transcript types: “wild‐type” BCR‐ABL without point mutations and truncated transcript with combination of del. c.1086‐1270 and mutation c.893T>G (p.L298R). Dasatinib therapy (140 mg/d) was initiated but discontinued after 3 years because of significant thrombocytopenia (15.4 × 10^9^ platelets/dL) and absence of molecular and cytogenetic (after first month of treatment—CyR = 31%, BCR‐ABL/ABL = 56.84%; after 3 years—CyR = 100%, BCR‐ABL/ABL = 125.39%).

**Figure 1:**
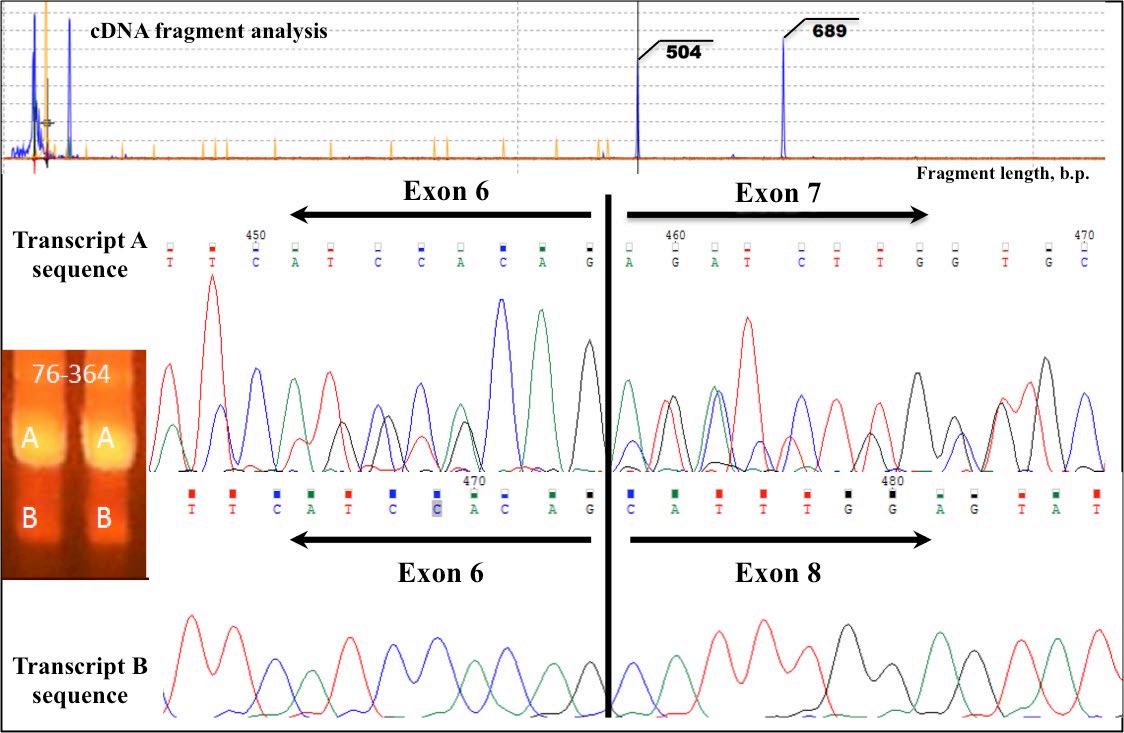
Detection of BCR‐ABL del. c.1086‐1270 by cDNA electrophoresis, fragment analysis and direct Sanger sequencing in a 2016 blood sample.

**Figure 2:**
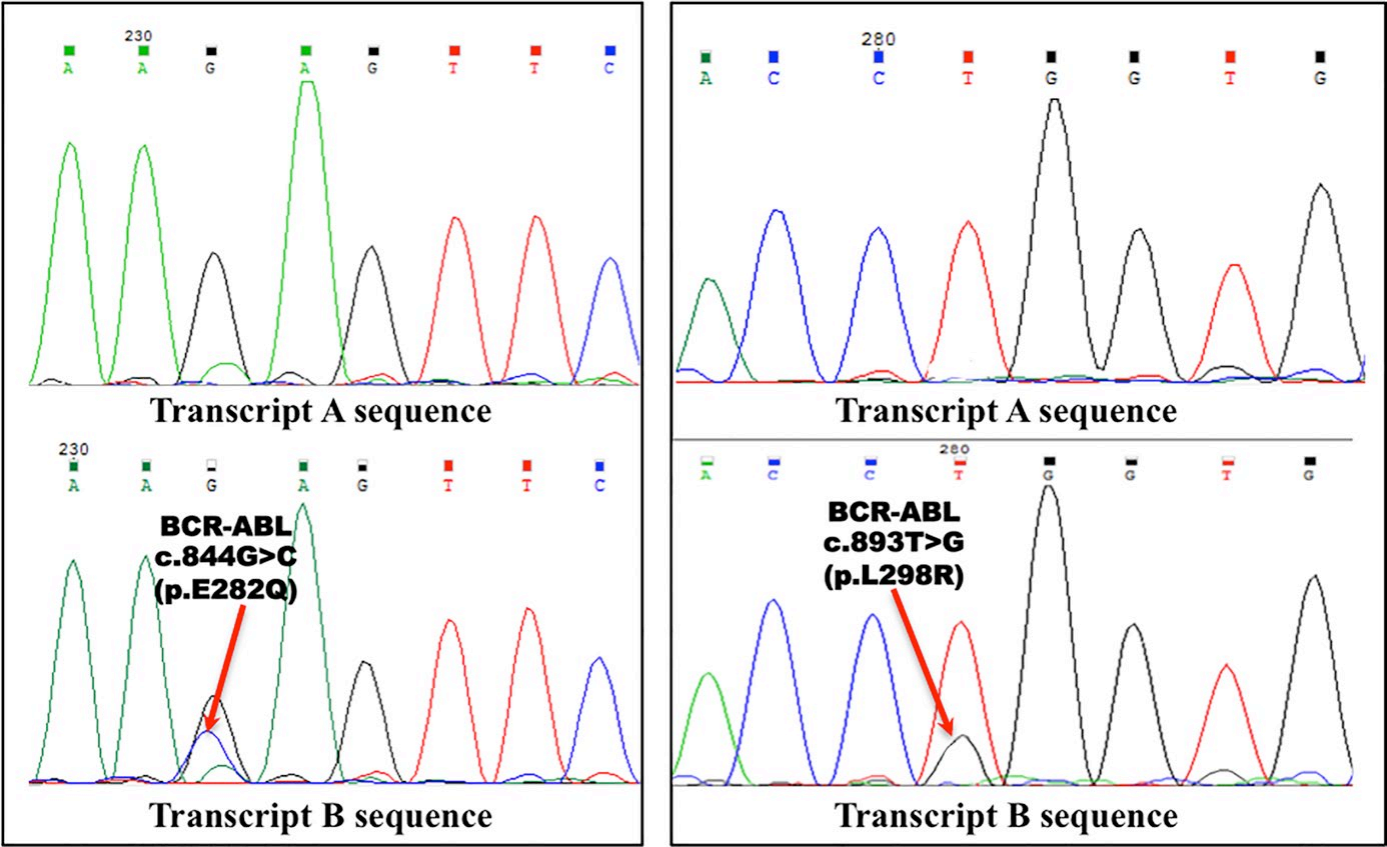
Continuation of cDNA sequence in a 2016 blood sample. Point mutations is absent in the transcript A (without deletion) but detected in the truncated transcript B.

In this way, it was decided to conduct fragment analysis and direct Sanger sequencing to identify point mutations. In 2016, cDNA fragment analysis has also detected two transcript types, and after their sequencing, I found that truncated transcript with del. c.1086‐1270 (Figure 1) and c.893T>G (p.L298R) has acquired a novel mutation c.844G>C p.E282Q which has not been described so far. Interestingly, point mutations were absent in normal length BCR‐ABL transcript (Figure 2).

According to these data, it was decided to offer the patient to participate in a Phase I clinical trial of a novel third generation BCR‐ABL TKI PF‐114 mesylate. Initial dose was 50 mg/d for the first month and now she receives dose 300 mg/d.

Next I decided to estimate the frequency of occurrence of exon 7 deletion among the patients of our center. A total of 33 male and 49 female CML patients (age 24‐80) with BCR‐ABL transcript level >0.1% were included in the study. Initial screening for deletions was performed by cDNA fragment analysis (Applied Biosystems 3130). BCR‐ABL del. c.1086‐1270 was estimated by nested PCR followed by Sanger sequencing. Deletion was found in 32 patients (39%). Fifteen of 32 (47%) patients with deletion were TKI sensitive and did not have additional point mutations while 17 (53%) were TKI resistant. All patients in the TKI‐resistant group had a history of proven resistance to at least one inhibitor, while 12 of 17 these patients did not have point mutations associated with resistance, and in 5 other patients, the following mutations were detected only in the deleted transcript (transcript with del. c.1086‐1270): F317V, F317L, E282Q, M351T, T315I, while they were absent in normal length transcripts.

## 3 DISCUSSION

I reported a case which can change our understanding of the pathogenic role of BCR‐ABL transcript with del. c.1086‐1270. This deletion was first described by Curvo et al^3^ in 2008, who suggested that this mutation appears as a result of alternative splicing and may be one of the causes of TKI resistance. However, Gaillard et al^4^ in 2010 showed that the frequency of deletion occurrence is not statistically different in the groups of drug‐resistant and sensitive patients. Moreover, they detected ABL1 del. c.1086‐1270 in healthy people without Ph‐chromosome. Computer modeling, which was performed by Meggyesi et al^5^ in 2012, showed that truncated transcript could produce only protein without kinase activity because of the disruption of ATP‐binding site. These facts are in good accordance with alternative splicing theory.

In 2016, a group of Indian researchers^6^ conducted a large screening study (385 resistant patients) that revealed a statistically significant relationship between deletion and TKI resistance. Our screening study showed that the deletion is detected approximately equally in sensitive and TKI‐resistant patients (47% and 53%, respectively); this fact is consistent with the alternative splicing theory. Thus, the pathogenic role of BCR‐ABL transcript with del. c.1086‐1270 on TKI resistance in CML is still controversial.

It should be noted that Berman et al^7^ in their study of relationship between TKI resistance and another possible BCR‐ABL splice variant BCR‐ABL1^35INS^ suggested that a truncated protein can heterodimerize with normal kinase and change its conformation. Similar mechanism was proved for splice variant of serine‐threonine kinase BRAF,^8^ which can cause resistance to vemurafenib.

**Figure 3:**
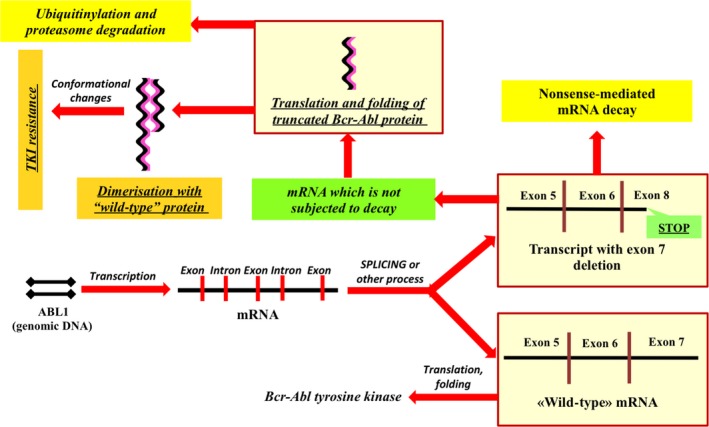
Possible mechanism of BCR‐ABL transcript with deletion of exon 7 participation in the formation of TKI resistance. Truncated tyrosine kinase molecules, which have not undergone proteasomal degradation, heterodimerize with “wild‐type” Bcr‐Abl proteins. This process can cause conformational changes that can lead to a disruption of the inhibitors binding to the ATP‐binding site of “wild‐type” Bcr‐Abl tyrosine kinases.

In this study, I report a case of resistance to first and second TKI generations which is caused by BCR‐ABL transcript with del. c.1086‐1270 and two point mutations (p.E282Q and p.L298R). Interestingly, these mutations were absent in “wild‐type” BCR‐ABL transcript. This fact strongly contradicts the hypothesis that del. c.1086‐1270 could be generated by alternative splicing. Moreover, this observation may indicate that the transcript with exon 7 deletion plays an important role in TKI resistance formation in this particular patient. I suggested that dimerization can explain the molecular mechanism in this case of resistance (Figure 3). Even if a truncated transcript does not directly participate in the formation of resistance, it can play a trigger role and enhance genomic instability in malignant cells and cause the appearance of combined point mutations.

Here, I also reported a novel BCR‐ABL point mutation c.844G˃C (p.E282Q) which is situated in special motif for interaction with SH3‐containing signal transduction proteins. I do not know whether this novel mutation plays a significant role in the formation of TKI resistance, additional studies on a large number of patients are needed.

## CONFLICT OF INTEREST

None declared.

## AUTHORSHIP

IM: discovered a novel point mutation and identified its combination with the BCR‐ABL exon 7 deletion. He then compiled a study plan for screening patients for the exon 7 deletion and performed all the laboratory procedures followed by the writing of this article.
